# How do people who use opioids express their qualities and capacities? An assessment of attitudes, behaviors, and opportunities

**DOI:** 10.1186/s12954-024-00981-4

**Published:** 2024-04-08

**Authors:** Jerel M. Ezell, Mai T. Pho, Elinor Simek, Babatunde P. Ajayi, Netra Shetty, Suzan M. Walters

**Affiliations:** 1 Community Health Sciences, Berkeley Center for Cultural Humility, School of Public Health, University of California Berkeley, Berkeley, CA USA; 2 Berkeley Center for Cultural Humility, University of California Berkeley, Berkeley, CA USA; 3 Department of Medicine, Section of Infectious Diseases and Global Health, University of Chicago Medicine, Chicago, IL USA; 4 Biology and Society, Cornell University, Ithaca, NY USA; 5 Department of Population Health at NYU Grossman School of Medicine, New York, NY USA

**Keywords:** Interventions, Opioid use, Social psychology, Harm reduction, Strengths-based

## Abstract

People who nonmedically use drugs (PWUD) face intricate social issues that suppress self-actualization, communal integration, and overall health and wellness. “Strengths-based” approaches, an under-used pedagogy and practice in addiction medicine, underscore the significance of identifying and recognizing the inherent and acquired skills, attributes, and capacities of PWUD. A strengths-based approach engenders client affirmation and improves their capacity to reduce drug use-related harms by leveraging existing capabilities. Exploring this paradigm, we conducted and analyzed interviews with 46 PWUD who were clients at syringe services programs in New York City and rural southern Illinois, two areas with elevated rates of opioid-related morbidity and mortality, to assess respondents’ perceived strengths. We located two primary thematic modalities in which strengths-based ethos is expressed: individuals (1) being and advocate and resource for harm reduction knowledge and practices and (2) engaging in acts of continuous self-actualization. These dynamics demonstrate PWUD strengths populating and manifesting in complex ways that both affirm and challenge humanist and biomedical notions of individual agency, as PWUD refract enacted, anticipated, and perceived stigmas. In conclusion, programs that blend evidence-based, systems-level interventions on drug use stigma and disenfranchisement with meso and micro-level strengths-based interventions that affirm and leverage personal identity, decision-making capacity, and endemic knowledge may help disrupt health promotion cleavages among PWUD.

## Introduction

People who nonmedically use drugs (PWUD) face persistent, overlapping challenges in obtaining quality healthcare and housing, acquiring and maintaining employment, and avoiding law enforcement encounters [1–4]. Many of these difficulties emanate from social stigmas and the drug criminalization *ethos* of the global legal and carceral system. This stigma oftentimes is multi-dimensional and is expressed in intricate, iterative ways. In terms of the psychosocial tableau of PWUD, *enacted* stigma refers to actual instances of discrimination or othering that is caused by their drug use (or outgrowths/associated behaviors); *anticipated* stigma involves the expectation of forthcoming/future discrimination or othering due to their drug use; and *perceived* stigma pertains to the individual’s perception of extant (negative) societal attitudes and stereotypes surrounding their drug use [5, 6]. Stigma fuels clinician and provider bias in treatment, lowers patient self-esteem, and leads patients to disengage from healthcare and other forms of institutional and community-level support [7].

Energized by binary belief systems regarding “right” and “wrong” behavior, these processes of social and ultimately political and economic marginalization generate a broad cascade of challenges for PWUD in their efforts to achieve and maintain a quality standard of living. Further, beyond negative impacts on general health, this structural oppression creates dire downstream impacts on PWUD’s families and communities, particularly those that are Black or Latinx and/or those that are low-income, in view of their already diminished social and political capital [8–10]. Collectively, these dynamics call attention to the potentially pronounced role that social perception research can play in establishing pedagogical models for discussing PWUD and approaches to clinically engaging PWUD.

Thoughtful and consistent application of strengths-based approaches has the potential to bolster myriad pedagogical dimensions in addiction medicine and counseling, including in engagements with PWUD from marginalized groups, with those experiencing co-occurring mental illness, and with those who are (or were formerly) incarcerated. Strengths-based approaches, particularly when they are guided or supported by individuals with lived experience, have been found to improve engagement in substance use treatment and to reduce “hard” and dangerous forms of drug use, such as injection drug use (IDU) and equipment sharing [11–13]. In contrast, deficits-based approaches, modalities that give primacy to and stress individuals’ weaknesses and limitations, have the dual effect of stigmatizing these populations *and* failing to incorporate their inherent or “would be” strengths into the formulation of interventions [14, 15]. With this in mind, Guo and Tsui [16] describe four key guiding epistemics for deriving strengths-based forms of epistemology and praxis:

1. *What is the difference between service users’ behaviour and the behaviour of the dominant class?*
2. *What is the relationship between service users’ ‘habitus’ (Bourdieu, 1990) and their social position? Why do they follow certain routines? Are there no other ways of proceeding?*
3. *What resources (i.e., social capital, personal capital and symbolic capital) do disadvantaged people possess that will enable them to change their lifestyle?*
4. *What kinds of strategies do disadvantaged people employ to further their interests and resist exploitation?*

Strengths-based frameworks in addiction medicine can leverage a patient’s native strengths and resiliency potential to foster more carefully conceived, tailored, and delivered forms of care. To this end, the contemporary framing of opioid misuse starkly differs from that of (crack) cocaine, marijuana, and methamphetamine misuse; this is due to the generally wider racial, ethnic, and class-related aperture of the opioid epidemic [17–21]. In other words, it is not limited to low-income, historically underserved populations, leading to subsequent variation in how opioid use is perceived, policed, and clinically mitigated according to these factors [8, 22]. A recent assessment of a representative sample of U.S. adults found mid-levels of stigma toward people with opioid use disorder (OUD), with disregard for OUD as a “valid” medical condition explaining most of the variation, highlighting a doubtful and ultimately negative impression of the capacity of PWUD [23]. Along these lines, research has assessed specific socioemotional resources and technical and relational skills that PWUD have pre and post-opioid initiation, but there has been limited assessment of how these dynamics manifest and the extent to which they are legible to the user and their networks and constitute elements of scalable interventions [24]. Specifically, there has been limited contextualization of how these attributes can be augmented to improve PWUD outcomes vis-à-vis treatment adherence, community integration, etc., a gap that the present analysis fills.

Given the cultural embeddedness of drug use stigma and the devaluation and discrimination that it produces [7], PWUD may be uniquely affirmed and motivated by strengths-based paradigms in ways that directly reduce their risk of drug-related harms. These harms most formatively includes overdose, but extend to associated sexual risks and downstream challenges to accessing social and health resources [25–28]. However, to date, there has not been an empirical characterization of intersections and opportunities between and within these paradigms. Here, we outline and analyze perspectives captured in interviews with clients from a syringe services program (SSP) in New York City, New York, and an SSP in rural southern Illinois, two areas with pronounced opioid-related morbidity and mortality rates.

## Methods

As part of a broader research initiative focused on the social epidemiology of nonmedical opioid use and drug injection and ways of enhancing access to harm reduction services and drug use treatments such as buprenorphine, we conducted interviews between July 2018 and October 2019 with clients at two geographically distinctive SSPs to enable a comparative analysis. One SSP was in an urban location, New York City, New York (hereafter referred to as “Ridge”), and the other was in a rural region in southern Illinois (hereafter referred to as “Anchor”). Anchor, a mobile SSP that is located in a community that borders Indiana, has key differences from Ridge, a stationary physical site, with the Anchor community having comparatively limited public transportation, lower-paying jobs, fragmented healthcare and drug use treatment services, and deeper institutional antagonism towards harm reduction, although syringe services programs are not legally prohibited in the state of Illinois [29].

In recent years, the broader southern Illinois region has seen outsized and persistent increases in opioid and drug injection-related morbidity and mortality [30, 31]. New York City, in turn, has recently experienced an increase in overdose-related deaths associated with synthetic opioids like fentanyl and fentanyl analogues [32]. Each area has also seen a surge in morbidity and mortality associated with polydrug use (e.g., intentional and unintentional combining of opioids with methamphetamines, (crack) cocaine, etc.) [33–35]. Of note, “Good Samaritan” laws (e.g., for reporting overdoses) exist in both Illinois and New York. However, these laws do not directly preclude harassment or other forms of disenfranchisement of the reporting individuals by law enforcement or their collaborators [36].

### Study overview and interview participation criteria

Guided by inductive processes, this present study endeavored to accomplish two goals: (1) to identify and characterize how PWUD at Ridge and Anchor interpret their skills, assets, attributes, capacities, and their expectations/goals in consideration of their opioid/drug injection initiation and continuation patterns and (2) to identify various individual-level strengths in this population that may be conducive to the development of more evidence-based, personalized, and culturally humble interventions for PWUD [37], with the ability to contextualize potential geographic variation. Given the complex and fluid ways in which these dynamics may be rendered, qualitative interview methods are of especially high utility. Interview questions included queries such as the following:

- *Please tell me about any experiences you have had using... [ask for each as appropriate: using pain pills/using heroin/injecting drugs; respectively].”*
- *Please tell me about the reasons you began using... [ask for each as appropriate: using pain pills/using heroin/injecting drugs; respectively].*
- *“Please tell me about your most significant experience with someone else overdosing? [If unclear: In other words, the experience (of someone overdosing) that affected you the most?]”*
- *“Please discuss with me about any times you went to a clinic or got treatment that could help you with drug use? (e.g., detox center? recovery center? Primary care office?)”*

Three interviewers conducted semi-structured in-person interviews with PWUD clients at Ridge and Anchor. One of the three interviewers had lived experience. The interviewers were taught specific culturally responsive approaches that emphasized patience and non-judgment in engaging PWUD respondents [38]. To be eligible to participate in the interviews, clients had to be at least 18 years old and have used any opioid “nonmedically” (i.e., without clinical guidance/recommendation) by any route of administration or injected any drug/IDU (opioid or non-opioid) in the last 30 days. Participants were recruited via outreach from SSP partner staff and members of our research team and later via referral-based sampling [39, 40]. The interviews, which took place in-person at the SSP site/mobile location, lasted between approximately 45 min and two hours and were audio-recorded. The audio files were then transcribed by a professional transcription company and reviewed and cleaned before formal qualitative assessment. Human subjects approval was obtained from the University of Chicago and Southern Illinois University. Before participation, written informed consent was obtained from all participants. Participants were compensated $40 for participating in the interview.

### Qualitative analysis procedures

Following each interview, the interviewer created an observational memo, organizing and characterizing formative and emergent impressions and perspectives. In addition to capturing the contextual aspects of the interview as part of “thick description” [41], a specific focus was placed on contextualizations related to the personality, dispositions, and orientations of the respondent. This primary analytic focus was oriented around understanding the qualities, expectations, and goals of PWUD inside and outside of the context of their drug use (i.e., *before*, *during*, and *post any drug* initiation/relapse periods). To guide the analysis, we assessed respondents’ commentary through the lens of the previously iterated strengths-based epistemics posed by Guo and Tsui [16], emphasizing acts of individualistic expression, resilience, and resistance.

Via an inductive process, we assessed PWUDs’ explicit and implied strengths in their pursuit of a higher quality of life (for themselves and others). To aid in category creation, we loosely defined ‘strengths’ in the context of social, political, recovering, and cultural health capital [42–45]. *Capital* as a metric here was inclusive of any skills, knowledge, attributes, or resources vis-à-vis their agency and capacity in feeling affirmed, safe, contented, and able to successfully engage in interpersonal relationships, healthcare, employment, and so forth. We further categorized capital along the previously iterated axes of enacted, perceived, and anticipated stigmas.

The interview data were assessed using modified grounded theory principles aimed at deeper contextualization of strengths-based typologies. This was supplemented by a sequential process of constant comparative methods, namely in view of potential spatial differences (i.e., urban vs. rural) [46, 47]. To begin the process, several conceptual codebooks were created, leveraging central thematic dimensions from the interview guide. Three coders then independently coded five of the same transcripts to identify core themes/subthemes and worked towards consistency in application approaches in the subsequent transcripts. As part of this reflexive process, additional codes were created and appended to address new constructs, with strengths-based typologies emerging with regularity.

Qualitative data processing and analysis were conducted using Dedoose version 8.3.21 and ATLAS Ti (v.9) to align with coders’ preferences. As a final step meant to better calibrate our interpretations and enhance the trustworthiness of the data, we carried out member-checking with non-participating PWUD familiar with the drug use milieus being explored and the various social and medical needs of PWUD [48], finding consistency with our baseline interpretations.

## Key findings

Overall, 46 individuals participated, including 24 PWUD clients at Ridge and 22 at Anchor. Complete sample traits are outlined in Table 1. In brief, the mean age of PWUD at both Ridge and Anchor was 38 years old (range 25–60 years old). At Anchor, 12 men and 11 women were interviewed (and one participant did not specify their gender). At Ridge, 14 men and 8 women were interviewed. Overall, 20 (83.3%) respondents at Ridge identified as White, two (8.3%) identified as Latino/Hispanic, and two (8.3%) identified as Black. At Anchor, 19 (86.4%) respondents identified as White, two (9.1%) as Black, and one (4.5%) as Native American. Across both sites, most respondents were either never married (41.3%) or were divorced (19.6%).

**Table 1:** Overview of PWUD Respondent Demographics (46)

	Ridge: New York City (n = 24)	Anchor: Rural Southern Illinois (n = 22)	Total
Mean age (range)	38 (26–60)	38 (25–60)	38 (25–60)
Gender			
Men	12 (50.0%)	14 (63.6%)	26 (56.5%)
Women	11 (45.8%)	8 (36.4%)	19 (41.3%)
Missing	1 (4.2%)	0 (0.0%)	1 (2.2%)
Primary race/ethnicity			
White	20 (83.3%)	19 (86.4%)	39 (84.5%)
Black or African American	2 (8.3%)	2 (9.1%)	4 (8.7%)
Latino/Hispanic	2 (8.3%)	0 (0.0%)	2 (4.3%)
Native American	0 (0.0%)	1 (4.5%)	1 (2.2%)
Marital status			
Married	1 (4.2%)	3 (13.6%)	4 (8.7%)
Widowed	1 (4.2%)	2 (9.1%)	3 (6.5%)
Divorced	6 (25.0%)	3 (13.6%)	9 (19.6%)
Separated	2 (8.3%)	3 (13.6%)	4 (8.7%)
Never Married	8 (33.3%)	11 (50.0%)	19 (41.3%)
Living with Partner	3 (12.5%)	0 (0.0%)	3 (6.5%)
Missing	3 (12.5%)	0 (0.0%)	3 (2.2%)
Education			
Less than High School	1 (4.2%)	1 (4.5%)	2 (4.3%)
High school or GED	10 (41.7)	11 (50.0%)	21 (45.7%)
Associate or Trade School	7 (29.2%)	3 (13.6%)	10 (21.7%)
Some College	3 (12.5%)	7 (31.8%)	10 (21.7%)
Missing	3 (12.5%)	0 (0.0%)	3 (6.5%)
Sexual Orientation			
Straight/Heterosexual	19 (79.2%)	14 (63.6%)	33 (71.7%)
Lesbian or Gay	0 (0.0%)	4 (18.2%)	4 (8.7%)
Bisexual	4 (16.7%)	2 (9.1%)	6 (13.0%)
Don’t know or not sure	0 (0.0%)	2 (9.1%)	2 (4.3%)
Missing	1 (4.2%)	0 (0.0%)	1 (2.2%)

In the following sections, we present emergent themes from the analysis. Themes are categorized in consideration of Guo and Tsui’s strengths-based epistemics [16] and our broader focus on exploring PWUD attitudes and behaviors towards their personal development and identity in a potentially spatially stratified context. Against this analytic backdrop, two salient themes emerged vis-à-vis PWUD strengths: (1) Being an Advocate and Resource for Harm Reduction Knowledge and Practices and (2) Engaging in Acts of Continuous Self-Actualization. Each theme had multiple subthemes that scaffolded into theoretical or applied operationalizations. Ahead, we use pseudonyms to relay PWUD’s comments.

Notably, while we endeavored to describe and contextualize geographic differences in respondents' perspectives and experiences, we did not locate substantial differences based on the site, suggesting a kind of universality and cohesion in PWUD experiences and self-reflections across space.

### Being an advocate and resource for harm reduction knowledge and practices 

One of the more pronounced and affirming roles that PWUD played was effectively serving as advocates and resources for various kinds of harm reduction knowledge/expertise and practices, communicating and manifesting core principles related to safe drug use and injection practices (e.g., helping during active overdoses, providing sterile injection equipment, etc.) and safer sexual practices (e.g., using contraceptives, getting tested for STIs, etc.). A central component of the advocate role was facilitating knowledge about risks, but more broadly involved PWUD acting as risk mediators or “intraventionists” for their peers, facilitating “prevention activities that are conducted and sustained through processes within communities themselves” ([49]: 250).

#### Preventing and mitigating peers’ drug overdoses

PWUD frequently discussed their roles as “guardians,” mediators in preventing their PWUD peers from overdosing. Tatum, a 29-year-old Black man client at Ridge, explains a scenario where this occurred:

I have experience with fentanyl. That's the drug that's killing a lot of dope fiends right now, [and] I saved two people’s lives because they was fucking with (using) fentanyl. One little man (friend) of mine’s wound up doing too much dope, and he was going to, what you call that, [have] an epileptic attack? Yeah, at the same time that he was overdosing. So, I had to hit him with the Narcan and hold him down so he doesn't go through a seizure episode. […] I saved his life. He thanked me for that, but he was fucked up because he shouldn't have been doing all that shit.

In discussing overdoses, Ash, a 48-year-old Latina client at Ridge, explains a similar scenario, describing her recognition of the severity of a younger peer’s overdose and her sense of obligation:

One time, this young kid ... he took like 10 “sticks” (units of drugs). Why would you take 10 sticks? He… was falling. I was like, ‘You know what? Why don't you go [to the hospital] with the guys that sold you all those freaking pills…’ ‘Please, please, can you take me?’ I didn't want to say ‘No,’ because I knew how really bad he was. I said to myself, ‘God, if I let him go and I hear in the news that he fell onto the train tracks or something, I'm going to feel really bad.’

Continuing this story, Ash articulates how certain nurturing, “maternal” instincts kicked in for her in the context of otherwise latent Good Samaritan laws and how she, as a member of PWUD population, felt indirectly bonded to the overdosing young man. Ash implied that these instincts were necessary to overcome the acute embarrassment and othering (anticipated stigma) that she would feel for having to be seen with an overdosing individual:

But what I told him was, ‘I'm going to bring you to the front of [name of street]… But I'm not going in with you. I'm not going to get embarrassed to be with you because I'm embarrassed.’ … I don't want to judge no one because I'm not a judger. I am not God to judge. If I could help somebody, that's what I'm here for… I'm a nurturing person, like I'm a mother also so I'm the nurturing type. I would never let somebody go and I know they're not feeling well, or whatever. I try to do my best to help my... *people that are like me*. You know what I mean?

In several instances, PWUD discussed situations where they were able to directly impart knowledge on the risks associated with certain drugs, or certain types of drug use, to a peer, at times even enrolling themselves as drug “testers.” Hayden, a 45-year-old White man who was an Anchor client, elaborates on this role:

I ‘guinea pig’ stuff for a reason—because I’m more careful than most people. Most of the drug users and most of my friends are aware of it. They’re happy to let me get some for free or whatever. They’re like, ‘Oh yeah, [Hayden] will know, and he’ll tell you and he won’t lie to you and he also won’t die on you,’ although it took some overdoses myself to get to that point… That’s why people do stupid shit because they don’t have the information. There is no such thing as too much information… not knowing how to compartmentalize that information, that can be a problem… as long as you have Narcan, that’s all you need to fucking have.

Along these lines, being an advocate (and standard-bearer) required a grounded form of “endemic” knowledge gained by first or secondhand experience and an associated calculated awareness of risks associated with drug use. With this in mind, PWUD discussed the importance of having boundaries in terms of where they obtained their drugs and, also, who they “exposed” their personal drug use to. As Hayden, who also sold drugs, adds:

I decided if I’m going to use hard drugs—and I tried them and I was like, ‘These are enjoyable’—I would do research. I wouldn’t just use blindly and randomly. [And] I’m not selling to you and you turn around and give it to this little 12-year-old boy […] People say there’s no responsible way to do damn drugs. But I’m safe as much as I can be around it. Like, I live in my dad’s house and he likes smoking it. I don’t even want to smoke it because my son is in that house.

Commentary such as this highlights how some PWUD may leverage certain situational skills, like the ability to do research in the absence of clarity from providers or other knowledge-holding professionals—acting as a kind of “citizen scientist” [50] to discern risks. This posture allowed PWUD to gauge and navigate the potential risks for themselves and those, such as other PWUD, whom they interface with. This commentary likewise leans into the notion of perceived stigma, pushing back on the general public’s presumptions that PWUD’s drug use decisions—as far as volume, location of use, etc.—are tactically unguided and socially reckless.

#### Hazard-proofing drug injection practices

Multiple respondents indicated that they were aware of individuals within their communities who engaged in IDU and shared needles, a risk factor for contracting HIV and hepatitis C [51, 52], and several respondents indicated that they had shared needles at least once at some point in the past but did not continue to do so. To this end, respondents highlighted different ways that they attempted to reduce the likelihood of them or their peers using non-sterile needles. One means of doing this was by personally distributing sterile injection equipment. Comparatively speaking, women in our sample were more likely than men to discuss and emphasize the importance of this role. This gendered difference suggests that women may have a higher propensity to recognize and actively participate in (and advocate for) harm reduction efforts, such as using sterile injection equipment. Describing her particular social network, Lane, a 60-year-old White woman who was a client at Anchor, says:

Oh yeah, [my peers] share needles. They call them ‘hand-me-downs’… I was watching motherfuckers hit themselves two or three times yesterday… When you’re around a bunch of bangers and you’re the only one with clean needles, I’ll let them use mine after I’m done. They know I’m clean. I’m Hepatitis-free, AIDS-free. I get tested every, probably, six months.

Hunter, a 31-year-old White woman client at Anchor—who similarly indicated that she was open to, and in fact, eager to share *‘clean’* (i.e., new/sterilized) needles—indicates that the multiple peers who shared needles were a potent motivation for her to be a sterile injection equipment advocate and resource. Hunter shares the following:

I share clean (sterile) needles with anybody… I made it real clear that I’m real sick of my friends fucking shooting up with dirty needles, and dull needles. And there’s no reason for it. They can come to me. I’ll give them my last needle. I don’t give a fuck… Like I said, that was the original purpose, you know? I’ve always been against [sharing needles].

As was the case in acting as an intraventionist for PWUD peers who might be at risk for overdosing, PWUD also served as a knowledge resource for equipment-sharing in a more health education-centric manner. Discussing a friend who was what she described as a “mild” user of drugs, Jai, a 31-year-old White woman client at Anchor, explains, “I have a friend, she just drinks. She don’t really do drugs. We’re friends and everything, she doesn’t do anything, but she doesn’t like the fact that [an SSP] delivers me syringes when I want and when I need.”

Elaborating on how she educated her skeptical friend on the function and value of SSPs and thus parrying this enacted stigma, Jai adds that being given syringes “…sounds like encouragement [to my friend]… but it’s not. I said [to my friend], ‘No. They're trying to prevent us [from not being] safe and clean."

Other PWUD discussed the importance of closely monitoring their injection equipment inventory to prevent harm to themselves and their loved ones (e.g., via accidental “sticks”), again contrasting with the hedonism frame often linked to PWUD [53]. Rory, a 41-year-old Black woman client at Ridge who had recently been living with her mother and sister, reflects on the importance of watching how she handled her injection equipment:

Just until recently, I got my own place. Beforehand, [I did not inject] because I wasn't alone—a lot of the time, my mom was home [and] my sister [was]. I had to be careful how I did that, because I didn't realize this back years ago, but I was kind of sloppy with my things, and I'd drop [needles]. I've learned to not do that anymore. I think I would have a lot more, had I not lived with my mom full time… because I would be alone a lot.

Rory’s fear of harming her mother and sister—for instance, by having them step on or simply see her needles—drove her desire to be more conscientious in terms of how she handled her equipment. This perspective signals Rory’s subtle anticipation of the anger or stigma that might come from her family should they become fully aware of her (‘sloppy’) drug use behaviors.

#### Asserting agency and clout in one’s sexual dispositions and practices

Respondents expressed multiple ways in which they attempted to lower their risk of acquiring a sexually transmitted infection (STI) such as HIV. These respondents highlighted the sometimes-uncomfortable discussions that were had with prospective partners and how they had to sometimes suppress or disrupt sexual desires to maintain a healthy sexual profile [54]. Hayden (Ridge) talks about the practicality of maintaining his sexual health in terms of risk reduction, cost management, and access. When asked why he used condoms, he stresses that they are “everywhere… there’s a church that have them [and] a counselor’s office!” He walks us through a hypothetical exchange with a prospective sexual partner:

‘Hi, I’m *X*. You’re *Y*? I’ve got a condom.’ I’m pretty confident [in saying this]. I’ve tried to be careful. I’ve tried to be smart… I [said to one of] my female friends that I was sleeping with: ‘[Condoms are] fucking free.’ I don’t understand why people don’t use them. I don’t like them. It’s not as fun, but whatever. It’s better than fucking gonorrhea, I’m sure.

Along these lines, Barrett, a 50-year-old Black man who was a client at Ridge, described his newfound clout and tendency in recommending his prospective sexual partners get checked for STIs. As part of his introspection into this dynamic, Barrett explains a hypothetical encounter with a prospective sexual partner who is seemingly ambivalent about STI testing:

It's crazy that people will have sex with me, and I'm the one that says, ‘Let's go to the clinic, and get checked,’ but they're like, ‘Fuck it…’ ‘Like, what if I got something, lady? You're not worried about if I've got something to give you…I'm willing to go to the clinic to show you that I don't have AIDS; at least I don't have AIDS, before we fuck. And here it is that just... you don't care. You're not pushing me to the point where I'm pushing you, like, because technically without that test, I don't really even wanna have sex with you.’

Going on, Barrett explains “So, I scare away from a lot of those encounters… It's bugged out; that was gonna be one of my new pick-up lines, ‘Let's go to the clinic!’ And a lot of ladies are like, ‘No, we don't want to…’ So, what can you do? I don't understand that.” Given the generally higher rates of STIs among Black individuals owing to disproportionate structural risks [55, 56], such proactive STI-testing dispositions are especially important.

Belief and subscription to other evidence-based interventions meant to reduce sexual risk, like PrEP, provoked similar inklings, with respondents expressing desires to be forthcoming and decisive. When asked how his partner would respond if asked to take PrEP, Robin, a 43-year-old Black man who was a Ridge client, indicates, “They wouldn't have no choice. If I'm doing it, you're going to do it too because it's benefiting both of us. You ain’t putting nothing in your body that ain’t healthy or it's going to kill you—so, why not?”.

Similarly, Murphy, a 47-year-old White man client at Anchor who had HIV, emphasizes the importance of candor and notes his sexual partner preferences, explaining: “I’m very upfront with my test result of [being HIV] positive, but usually I either mess around (have sexual encounters) with somebody who is either on PrEP, who is taking it religiously, or someone who’s already [HIV] positive.”

Aligning with theory on intravention [49, 57, 58], these and other PWUD in our sample were apt to provide education and knowledge to peers on risks associated with various forms of drug use (specifically overdose and needle/syringe sharing) and sex, in the interest of preventing drug-related morbidity/mortality and STIs, respectively.

### Engaging in acts of continuous self-actualization

A primary focus for respondents was on being, or becoming, “better” people through iterative, flexible processes of continuous self-actualization. However, being ‘better’ did not always correspond to engaging in or successfully completing or partaking in treatment or a recovery program—cessation frequently being a point of emphasis and proposed “endgame” in the drug use intervention literature [59–62]. For example, entry into treatment was characterized by respondents as what-should-be a largely independent decision that did not need to be foisted upon them by institutions (e.g., courts or employers) or other external actors (e.g., romantic partners, family members, etc.). This paradigm of bettering oneself involved, first, processes of self-growth and productivity that involved constantly challenging and resetting out-group expectations on behavior and goals. Relatedly, respondents reified dynamics such as pleasure-seeking, resilience, and resistance as avenues for undoing intragroup and internalized stigma.

#### Pursuing self-growth and productivity

Respondents described a broad interest in self-growth and productivity uncoupled from convention, while nonetheless negotiating and managing expectations on the “right” forms of treatment and recovery as part of a continuous process of self-actualization and undoing stigma and avoiding (social) policing. Taylor, a 43-year-old White man client at Anchor who had recently been incarcerated, offers the following:

I am in the process of recovering. It’s a fight every day, don’t get me wrong. I still do Suboxones (a medication for OUD) which is an opiate. So, I still am in it, the addiction, to an extent. I’m not no better than nobody. I try to make better choices today than I used to. I don’t want to be in prison no more. I don’t want to go on parole for nothing. I just don’t want to deal with cops. I don’t want to pay fines.

Similar sentiments were shared at Ridge, illustrated vividly by Robin who deliberated on the boundaries of a “right” and a “wrong” form of treatment and recovery versus simply *being* as one wants (namely, using drugs how they desire). Robin, recognizing the extent to which this deliberation reinforces his positive philosophy on effort and ongoing, dynamic personal development, captures the perceived stigma of “inadequate” recovery:

I'm used to the feeling of using everyday with no breaking point… when you get clean for two weeks, you gotta go back and do all that ‘clean time’ over again. ‘I don't give a fuck if you're clean for one day. That's a good thing if you can stay clean one day.’ But I remember there was one day I couldn't stay clean. So now, every day I'm just moving, pressing that I can stay clean. If I can stay clean for one day, I can do another day. And I'll do another day, and then, before you know it, your days end up a week.

In this regard, the ability to set goals toward recovery was described as being greatly amplified by social support, which then reinforces PWUD strength. Zane, a 39-year-old White man client at Anchor, emphasizes this in discussing how impactful connections with loved ones are when one is in recovery. He also notes that the rehab he attends is far from his hometown, suggesting amplified barriers to service access and social support for clients at Anchor. Zane highlights the potent social support provided by his family, remarking that it:

…gives you something to reach for. ‘I got to do good this week. I do good this week, so I can tell them all the good things that I’ve done when they come to see my visit and I can get that little pat on the back that I’m needing.’ That what they need to do. They need to do more [rehabs] and they need to have more night sessions... They’re expecting because you’re a dope head, you don’t have a job. Well, people that used to be dope heads have jobs.

Along these lines, employment was cited by multiple respondents as a major source of satisfaction, fulfillment, or inspiration. Addison, a 36-year-old White man who was a client at Anchor, illustrates this in discussing his prior experience in nursing: “I loved it. I was the only boy in my class and… there was me and one other girl was in kind of a tie for the first in class… All I had ever known was nursing.”

At Ridge, employment was most directly cited as a source of pride and a mark of self-actualization that was or could be effectively uncoupled from one’s drug use status. Illustrating a desire for (financial) independence, multiple respondents regarded not having work as a source of shame and disappointment, as Malen, a 51-year-old Latino man client at Ridge who was interested in employment as a driver, shows: “Basically, I'm just on public assistance right now. Yeah, I'm not proud of that. But it's just a stepping-stone before I find myself a real job. I don't like to be living off the government. I like to have my own job.” Potentially highlighting a Latino cultural value associated with resistance to government interfaces and “dependence” [63], as a means of dispelling enacted stigma from own’s community, this finding highlights a potentially distinctive collectivist attitude that may push some PWUD away from macrostructural systems deemed oppressive or binding.

Further adding to this theme, employment served an intuitively practical function for PWUD at Ridge as well. This was especially the case for those who engaged in sporadic work or work in the informal economy, roles frequently occupied by PWUD due to their exclusion from traditional sources of employment as an enacted stigma (owing to them often having criminal records and/or a perception that they lack capability and/or reliability) [64]. Elliot, a 34-year-old Latino man client at Ridge who did odd jobs including landscape work and picking up consumer items for people, showcases this:

I've got to go to work. ‘You want a cup of coffee? You want a sandwich?’... And then, ‘go to my rowhouse that's fucking 200 years old and pull up the weeds in my fucking flower pots.’ I'll go do that and they'll pay me, so, I don't feel like I owe them something. Usually, they'll never give me a job that takes more than an hour because they know I'm a fucking drug addict.

Spencer, a 58-year-old White man who was a client at Anchor, stresses how this form of grit—and his status as an effectively “unbounded” unhoused person engaged in panhandling as a primary source of income—afforded him a type of freedom and empowerment not enjoyed by non-PWUD who are housed and have “formal” jobs. He speaks indignantly about perceived stigma, like Elliot, whilst highlighting more niche, endemic knowledge in articulating the fruits of the informal [street] economy [65]; this perhaps reflecting what was described by Hughes [66] as *dirty work* (by good people): “Me being in the street… roaming around, being a drug user, and everything, you know how much money I come across? ‘Alright. So, you're stuck at your little dead-end job for whatever you're making, because you chose that… I'm out there panhandling, and people are giving me hundreds.’”.

Respondents here illustrate a recognition that self-actualization could and often did need to occur outside of the context of their drug use and indeed occurred along a continuum. This finding is important for two reasons. First, it highlights the extent to which some PWUD regard themselves as “more free” and ascendant and their growth trajectories as more dynamic, rather than fixed. And second, it highlights the extent to which PWUD are compartmentalizing their identities to adjust to the salience of their drug-using preferences and economic constraints.

#### Undoing Intragroup and Internalized Stigma

Contrasting with the mainstream perspective of the PWUD “experience” as a monolithic one ruled by despair, deviance, and/or inactivity [67], one significant component ascribed here to the PWUD experience was finding pleasure being productive, and indeed drug use or recovery themselves being forms of production, and thus actively “undoing” intragroup and internalized stigma. Iterative stigmas ascribed to PWUD vis-à-vis social norms on what drug use should or should not be about and how extrinsically engaged PWUD are have particular import here. Previous research has documented the relational imposition of negative labels and dissociation *between* PWUD [68]. As Petra, a 25-year-old Latina client at Ridge, explains, “I used to tell myself, when I used to be [dope]sick, I was still myself. I came across a lot of drug addicts… actually, the one who got me to start injecting, when he wasn't injecting, he was a turtle. He didn't even laugh, smile… He was completely different…”.

Going on, illustrating that the life of PWUD can, in contrast, be pleasurable and balanced, even as one grapples with being dopesick and the prospect of being “outed” as a PWUD, Petra adds, speaking to fluid acts of compartmentalization:

Whether I'm sick or I'm not, I was always listening to music. I can always dance... Actually, music helps me when I'm sick. It helps me forget about it. I can still laugh. I can still be myself. I can still joke around. And that's one thing that has never changed about me throughout my whole journey, whether using or not using. That's why it was kind of not as hard as I thought it would be to maintain the double life.

Along these lines, another critical part of this process was PWUD recognizing themselves as equal to non-PWUD, feeling as though their lot in life, for better or worse, could be *anyone’s.* As Paige, a 31-year-old White woman client at Anchor, argues:

*Everybody* suffers from addiction, people that have been poorest of the poor and ... people that have millions of dollars in the bank, you know? And you all just ... everybody comes down to an equal level there. You know? We’re all just fucked up, trying to get better, you know? And I’ve met some really awesome people in rehab. Some of the best people I’ve ever met in my life [are in rehab].

Paige believes that while everyone is fundamentally ‘fucked up,’ everyone also has strengths, attributes, and the desire to continuously improve. As Barrett (Ridge) demonstrates, the feeling of difference, as a non-sober person as compared to a sober person, can accentuate PWUD’s sense of actually having more (intangible) strengths than non-PWUD. Using the hypothetical example of PWUD who could maintain a job as a CEO, he articulates the following:

I've been trying to stay sober here and there, because of what I'm going through, and people don't respect what they don't understand, and when they find out... whether it's like cocaine, heroin, crack, automatically, ‘Oh, you have to be no good.’ ‘But for 100 years, you didn't know I was smoking crack, and I was a CEO. And then, the next day, tomorrow, you find out I smoke crack, you don't want me to be CEO. But every 100 days before that, I was smoking crack, and shooting heroin, and all that shit for all those times you never knew nothing, and I still got my job done.’

Continuing, Barrett further notes, his comment imbued with anticipated stigma, “So, I try to stay sober now to prove them wrong, that I can accomplish everything… which doesn't make me any better than a drug addict, but it’s just shoving it into their noses… the ones that think they're better than us, because they're sober.”

While mostly focusing their comparisons on non-PWUD, respondents also framed their lives in comparison to those of other PWUD or, more acutely, to the notion of a stereotypical PWUD vis-à-vis tropes associated with unruliness, uncleanliness, etc. [8, 69]. These framings relayed simultaneous acts of identity maintenance and intragroup and internalized stigma management with institutions, other PWUD, and non-PWUD.

Continuing, respondents expressed mixed views on how various institutions, namely law enforcement, engaged with PWUD. Rather than regarding their positions on the criminalization of drugs as self-serving, respondents reflected orientations that were legally logical and frequently spoken of as a defense of pluralism and justice. As Zane (Anchor) explains, discussing his misgivings about drug criminalization laws:

Ever since I’ve been young, I’ve been told that not everything that the authorities tell you is true and that not all drugs are as bad as they say. While I understand my mother’s intentions and I agree with her—yes, she was correct—it also instilled in me hard distrust of authority that remains to this day. I think it’s a *healthy* distrust… it’s healthy because there should be oversight for anyone in a position of power or authority.

As Zane further highlights, such orientations are also a reflection of social and cultural health capital and individual/patient agency and clout [43]. Discussing a doctor who would not prescribe him Benzodiazepines unless he stopped smoking marijuana, Zane illustrates his self-advocacy skills:

He said, ‘Well, you’ve got to be clean first.’ ‘So, you’re telling me I have to go

through fucking panic attacks for a month for at least 30 days to get it out of my system. So, I have to suffer for a month for you to do your job? Uh-uh (negative).’… I treat the doctor like what he is—he is my employee. ‘You work for me. I come in here, you get paid because I’m here. You’re my employee. I don’t care if your taxpayer dollars pay for it or what.’

Commentary in this domain reflects intersecting efforts focused on identity maintenance and, more directly, on intragroup and internalized stigma management and agency-building aimed at controlling and managing expectations about personal outcomes, while also establishing expectations for a broad cast of *external* stakeholders—e.g., clinicians, courts, law enforcement, etc.—who are needed to ensure the safety and wellbeing of PWUD.

## Discussion

In this article, we captured and contextualized strengths-based paradigms that manifest in the attitudes and experiences of PWUD, including them being advocates and resources for harm reduction knowledge and practices and them engaging in acts of continuous self-actualization to achieve individual affirmation and counter multidimensional forms of stigma. Much of the respondent commentary here was linked to perceived and anticipated stigmas, which bridged to and were reified by enacted stigma in various everyday social interactions, employment, healthcare, and so forth. Of note, while we located some gendered patterns in responses, we did not find substantial differences in strengths-based paradigms (or lack thereof) in other domains, such as geography or race, potentially suggesting the presence of a formative experiential throughline. This finding may speak to the relative uniformity in how structures, particularly political and criminal justice institutions and popular media, establish and enforce particularly social psychological typologies around drug use.

Considering the highly relational aspect of drug use, our work punctuates the notion that PWUD are often mindful of how others may perceive and be impacted by their drug use. Indeed, PWUD here frequently demonstrated a keen capacity for empathy—indeed even more than they may receive—and for risk reduction inside and outside of their immediate habitus. These are tendencies that can be amplified through affirmational policy and intervention promoting deeper health literacy and capacity-building in this group.

Two vital paradigms that emerged here related to recovery and peer support. PWUD from both sites conceptualized recovery as an iterative, dynamic process and were critical of generalized tendencies to view recovery as a fixed endpoint [59–62]. Both groups denounced the hierarchical distinction between “clean” and “unclean,” emphasizing that continued effort toward recovery is, if desired, of equal import and utility. Along similar lines, PWUD in this sample highlighted the importance of peer support, which bolstered mental health, to recovery. Recovery and peer support are important components for mental health treatment [70, 71].

Providing both tangible resources and intangible social support, PWUD potentially increased the recovery capital of their peers through intravention [42, 49], with us further finding preliminary evidence that women were more comfortable with, or more accustomed to, harm reduction advocacy, knowledge-sharing, and resource support than men in the sample. This finding illumines the potentially gendered, networked nature of strengths-based dispositions [72], which may portend differential levels of intravention. Moreover, the observed gendered difference in harm reduction knowledge-sharing and practices may point to a heightened sense of “social responsibility” among women toward their social networks [73]. It is plausible that these women were socialized with expectations to exhibit traits such as empathy, compassion, and nurturing–traits all more commonly ascribed to women relative to men [74, 75]. Such findings illustrate the need for more research on the potential gendered differences in intravention among PWUD.

**Fig. 1:**
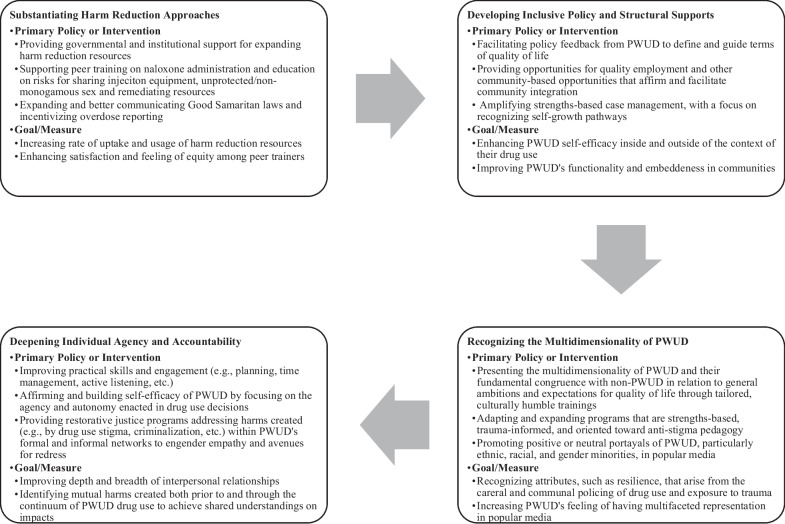
Strengths-based paradigms, policies, and therapeutic approaches for PWUD

Maton et al. outline several strategic goals for strengths-based pedagogy in clinical and therapeutic practice, including recognizing and building on existing individual, family, and community strengths, building new strengths in each, strengthening the larger social environments in which each is embedded, and engaging each in a strengths-based process of designing, implementing, and evaluating interventions ([14]: 6). Considering this framework, in Fig. 1, we summarize specific paradigms, policies, and therapeutic approaches to capitalize on the skills, attributes, and assets identified in this analysis. In brief, through the implementation of naloxone administration training and other risk education/reduction initiatives (particularly related to injection equipment sharing and having unprotected sex and concurrent sexual partners), SSPs can be utilized as a formative means of social, physical, and mental self-care. To this end, findings here heavily underscore the importance of deepened harm reduction policy given that many of the resources that PWUD cited as facilitating intravention (e.g., sterile needles/syringes, condoms, STI testing, naloxone kits, etc.) are provided for free by SSPs and other harm reduction-oriented organizations and governmental agencies.

Policy that allocates funding to harm reduction can broaden the scope of intravention to include PWUD who face systematic barriers to safe drug use and other health-promoting outcomes. For instance, because PWUD in rural areas (such as those from Anchor) are more spatially isolated from harm reduction organizations and healthcare more broadly, expanding access to harm reduction services can broaden PWUD intravention networks, increasing the recovery capital of PWUD within the local network [42, 76, 77].

Sustained harm reduction approaches can aid PWUD’s integration back into their communities via the cultivation of healthier living practices and more inclusive cultures, tasks that, due to social network compression, may otherwise be difficult in communities such as the rural ones presented here or in segregated areas [78, 79]. Moreover, by facilitating access to quality training and education opportunities and recognizing other avenues of self-acutalization that can be conveyed to prospective employers, practitioners can help bolster the self-efficacy of PWUDs in ways that also address their underlying social/economic vulnerabilities. Along these lines, strength-based pedagogy codifies that the broad ambitions and interests of PWUD are relatable to those of non-PWUD. Thus, strengths-based approaches can serve the dual purpose of developing clearer arcs of agency, clout, and accountability among PWUD while also helping practitioners develop the skills needed to further shape programs that address harms related to drug use.

With this in mind, in response to critiques about the difficulty of operationalizing and measuring strengths-based programs [24, 80], we briefly outline potential ways to assess the PWUD-specific goal or means to measure the extent to which the efforts are strengths-based and potentially efficacious.

Considering PWUD as individuals with endemic knowledge who are “insiders” in the most visceral sense, two interconnected layers of potential social welfare policy to leverage this insider status emerge. The first layer of policy should focus on putting PWUD in positions to provide direct “policy feedback,” in alignment with the aims of Community-Based Participatory Research (CBPR) and restorative justice [81, 82]. CBPR places community members (in this case, PWUD) in a position of influence often reserved in the realm of research for academics and policymakers. In shifting this paradigm, CBPR would allow PWUD to directly discuss their needs and envision research and policy that could address these needs against the backdrop of clearer acceptability. Further, restorative justice can help to ameliorate the often-tense relationships between PWUD and regulatory organizations such as law enforcement and the court system. This policy feedback should provide space to discuss PWUD needs vis-à-vis concerns regarding general healthcare, employment, housing, community supports, etc. The second layer of policy should focus on amplifying financial resources for PWUD, for example, by supporting peer education programs (made reimbursable through Medicaid, etc.) and augmenting existing harm reduction programming, including SSPs and safe consumption sites, as well as the distribution of naloxone, fentanyl testing strips, etc.

Importantly, our findings suggest that PWUD agency is intimately tied to capacity, with respondents here expressing deep levels of awareness of their place along the drug risk continuum; these dynamics potentially influencing feelings of self-efficacy, hence impacting overall health and wellness [7, 83]. This dynamic illumines the need for interventions and policies that affirm the multidimensionality of PWUD and that directly support their integration in communal settings. Furthermore, in considering client goals, it is essential to recognize whether they derive from PWUD or are directly or indirectly “superimposed” onto them by others. Strengths-based interventions with PWUD are most effective when they align themselves to the formative decision-making processes of the target population–this being the central axiom of CBPR [84–86]. By embracing the ethos of shared decision-making, these interventions can undercut  the tendency toward coerced responses to perceived, enacted, and anticipated stigmas [87].

Finally, our findings highlight several important implications for individual practitioners as well as agencies. Practitioners may benefit from leveraging PWUD peer connections to support recovery; as an example, clinicians can discuss a client’s safety plan in the event of overdose, encouraging clients to use drugs with peers rather than alone. Further, in alignment with cultural humility [88, 89], clinicians can practice self-reflection on potential biases related to engagement and recovery and allow clients to lead discussions on their expectations, prior experiences, and their personal recovery goals, which may not correspond to clinicians’ expectations or values. Finally, clinicians and agencies can provide institutional support for PWUD intravention by helping to thoughtfully integrate PWUDs’ “best practices,” in terms of approaches and messaging, into harm reduction programming and/or drug use treatment [90].

### Limitations of the study

There are some limitations to this analysis. First, we relied on qualitative data collected from PWUD clients who were actively engaged in harm reduction/SSPs. Hence, our findings may not be reflective of PWUD who are not engaged in harm reduction or SSPs—potentially due to not having access to these resources, being unaware of the existence of these resources, or simply being disinterested. Moreover, our focus on clients may have led to social desirability bias, insofar as respondents, as active SSP clients, may have felt compelled to convey positive impressions of SSPs and harm reduction more generally [91]. Nevertheless, we provide a window into the conceptualizations that may color the considerations of PWUD in high-risk urban and rural communities who are connected to resources that are vital to disrupting the opioid epidemic and can serve as knowledge, resource, and support conduits for PWUD who do not presently utilize SSPs.

Continuing, the overall sample was primarily White, and thus it is unclear how non-White PWUDs’ experiences may have corresponded to themes and subthemes that emerged from this work. Along these lines, respondent sentiments captured here likely have resonance with the more universal experience of marginalization of PWUD across multiple axes of social identity and environment and thus can inform strengths-based practice more generally. Furthermore, our work focuses on people who primarily used opioids or who injected drugs (opioids and non-opioids). Hence, the findings may not be applicable to people who primarily used other types of drugs.

Despite these limitations, results from this qualitative analysis provide evidence suggesting that strengths-based paradigms are highly valuable for PWUD both inside and outside of the context of their drug use. Indeed, social psychological contextualizations raised here illuminate that strengths-based paradigms are of special import precisely because they simultaneously accentuate the interstices in the “before”, “during,” and “after” phases of drug use. In turn, these paradigms can be disruptive to the net impact of enacted stigma and can reveal multiple levels of identity—vis-à-vis, class, gender, social network centrality, etc. To maximize the efficacy of therapeutic interventions and policies, it is imperative to integrate strengths-based modalities that specifically consider these diverse markers of social identity and embeddedness. Such approaches will provoke a unique reflexivity in both PWUD and those they may interact with, clinically and otherwise, thus ensuring more holistic, self-aware, and layered interactions.

## Data Availability

Not applicable.

## Abbreviations

IDU: injection drug
OUD: Opioid use disorder
PWUD: People who nonmedically use drugs
SSP: Syringe services program
STI: Sexually transmitted infection
